# Integrating maternal, newborn, child health and non-communicable disease care in the sustainable development goal era

**DOI:** 10.3389/fpubh.2023.1183712

**Published:** 2023-06-27

**Authors:** Svetlana Akselrod, Anshu Banerjee, Téa E. Collins, Shambhu Acharya, Nazira Artykova, Ian Askew, Nino Berdzuli, Sergey Diorditsa, Rudolf Eggers, Jill Farrington, Zsuzsanna Jakab, Carina Ferreira-Borges, Bente Mikkelsen, Natasha Azzopardi-Muscat, Victor Olsavszky, Kidong Park, Howard Sobel, Huong Tran, Melita Vujnovic, Martin Weber, Wilson Were, Nuhu Yaqub, Daria Berlina, Catherine L. Dunlop, Luke N. Allen

**Affiliations:** ^1^Global NCD Platform, World Health Organization, Geneva, Switzerland; ^2^Maternal, Newborn, Child and Adolescent Health, and Ageing, World Health Organization, Geneva, Switzerland; ^3^Country Strategy and Support, World Health Organization, Geneva, Switzerland; ^4^WHO European Region Country Office in Kyrgyzstan, Bishkek, Kyrgyzstan; ^5^Sexual and Reproductive Health, World Health Organization, Geneva, Switzerland; ^6^Division of Country Health Programmes, World Health Organization Regional Office for Europe, Copenhagen, Denmark; ^7^WHO Representative's Office, WHO European Region Country Office in Belarus, Minsk, City of Minsk, Belarus; ^8^Integrated Health Services, World Health Organization, Geneva, Switzerland; ^9^Deputy Director-General Office, World Health Organization, Geneva, Switzerland; ^10^NCD Department, World Health Organization, Geneva, Switzerland; ^11^Division of Country Health Policies and Systems, World Health Organization Regional Office for Europe, Copenhagen, Denmark; ^12^WHO European Region Tajikistan Country Office, Dushanbe, Tajikistan; ^13^Data, Strategy and Innovation Group, World Health Organization Regional Office for the Western Pacific, Manila, Philippines; ^14^WHO Regional Office for the Western Pacific Country Office in the Solomon Islands, Manila, Philippines; ^15^Division of Programmes for Disease Control, World Health Organization Regional Office for the Western Pacific, Manila, Philippines; ^16^WHO European Region Office for the Russian Federation, Moscow, Russia; ^17^Division of Country Health Policies and Systems, World Health Organization Regional Office for Europe Country Office in Greece, Copenhagen, Denmark; ^18^Child Health and Development, World Health Organization, Geneva, Switzerland; ^19^Institute of Metabolism and Systems Research, University of Birmingham, Birmingham, United Kingdom

**Keywords:** maternal health, newborn health, non-communicable disease (NCDs), child health, policy and guidelines

## Abstract

Noncommunicable diseases (NCDs) and maternal newborn and child health (MNCH) are two deeply intertwined health areas that have been artificially separated by global health policies, resource allocations and programming. Optimal MNCH care can provide a unique opportunity to screen for, prevent and manage early signs of NCDs developing in both the woman and the neonate. This paper considers how NCDs, NCD modifiable risk factors, and NCD metabolic risk factors impact MNCH. We argue that integrated management is essential, but this faces challenges that manifest across all levels of domestic health systems. Progress toward Sustainable Development targets requires joined-up action.

## Highlights

- Maternal, newborn, and child health (MNCH) is inextricably linked with noncommunicable diseases (NCDs), their risk factors, effective management, and outcomes. Noncommunicable diseases are currently one of the leading causes of death among women of reproductive age in many countries.
- Pregnancy and childbirth are often not prioritized in NCD policies and programming, and the NCD-related morbidity experienced during pregnancy do not routinely receive consideration by healthcare workers, nor do the NCD risk factors.
- Pre-existing NCDs and exposure of the woman and fetus to NCD-shared risk factors during pregnancy can increase the risk of miscarriage, stillbirth and maternal death. Increased risks to women and neonates from NCDs include premature birth, birth injuries, congenital malformations, and infant respiratory distress syndrome, contributing to the poor health of future generations.
- There is a need to strengthen and reorient health systems to enable the integration of NCD prevention, screening, diagnosis, and management as part of MNCH care programs.
- Ensuring the quality and consistency of health interventions when scaling up services to integrate NCD and MNCH care can be challenging at all levels. Existing barriers can be overcome by strong political will, intersectoral action and increased investments in the continuum of care across services and using a life course approach.

## Introduction

Maternal, newborn, and child health (MNCH) has been prioritised in the Millennium Development Goals (MDGs), the Sustainable Development Goals (SDGs), and other global health policy agendas, which—combined with increased funding and technological advances—has contributed to remarkable reductions in preventable mortality since 2000 (1). Yet, annually there are still nearly 300,000 maternal deaths worldwide, two million stillbirths and almost six million children die, mostly due to preventable causes (2–5).

Noncommunicable diseases (NCDs), including cardiovascular diseases, cancers, chronic respiratory diseases, and diabetes are collectively responsible for over 70% of all morbidity and mortality worldwide (3). Whilst NCDs were missing from the MDG targets, (1) they have become an urgent global health priority, reflected in the WHO 13^th^ General Programme of Work and Sustainable Development Goal (SDG) target 3.4 (6, 7).

MNCH and NCDs are inextricably linked. NCDs are one of the leading causes of death among women of childbearing age (15–49 years), and two-thirds of deaths among all women are due to NCDs (19 million deaths per year) (3, 8). High mortality due to NCDs among women aged over 50 years also suggests that many younger women are already at risk. Indeed, as of 2019, about two billion women aged 15–49 years were affected by NCDs and their shared risk factors globally (3, 8). This demonstrates the need for SDG target 3.4—a one-third reduction in premature mortality from NCDs and enhanced mental health by 2030 (9). Optimal MNCH care can provide a unique opportunity within the healthcare system to screen for, prevent and manage early signs of developing NCDs. However, women’s unique experiences of pregnancy and childbirth and their specific healthcare needs are often not prioritised in NCD policies, resource allocations, and programming.

The convergence of NCDs and MNCH agendas is a public health concern worldwide, especially in LMICs. For example, 90% of all diabetes cases are in LMICs (3) and the prevalence of perinatal depression is higher among women in LMICs than in high-income countries (HICs) (10). The agenda for addressing maternal mortality and morbidity has historically focused on direct obstetric causes, such as postpartum hemorrhage and pre-eclampsia. However, increasingly non-direct causes, including NCDs, are becoming the major burden in this population in a process deemed the “obstetric transition,” occurring in both LMICs and HICs (11). Despite significant overlap, NCDs and MNCH are often treated as completely separate entities in global health financing, planning, and programming (12). Integrated action across these domains can enable women and children to live long, healthy lives (13). This paper explains how NCD and their risk factors intersect with maternal neonatal and child health, the challenges of integration, and the pressing need for convergence in policymaking and clinical practice.

### NCDs during pregnancy and childbirth

The interplay and opportunities for integration between NCDs and MNCH are multifaceted—including a possibility for screening for those at risk in the pregnant population, early detection of NCDs, optimizing management of existing NCDs and addressing risk factors and exposures for future development of NCDs for both the woman and the fetus (13). Additionally, pregnancy is a unique time when underlying health conditions can be exacerbated and new conditions can develop (14). These affect the ongoing health of both the mother and child (13).

For pregnant women with existing chronic conditions, NCD-related morbidity experienced during and after pregnancy often does not receive adequate consideration by healthcare workers (2). NCDs can negatively affect both current and future pregnancies and may lead to developmental challenges in infancy and early childhood for the neonate, contributing to the poor health of future generations (13). As an example, pre-existing chronic conditions can increase the risk of spontaneous abortion and stillbirth, premature birth, birth injuries, congenital malformations, infant respiratory distress syndrome, and several other complications in the mother and newborn child (2).

The more common NCDs affecting pregnancies include diabetes, asthma, epilepsy, hypertension, and mental health conditions, presenting opportunities for the integration of MNCH and NCD care. Asthma affects 2–13% of pregnant women worldwide and can result in adverse pregnancy outcomes, particularly when not well-controlled, with increased risks of pre-eclampsia, gestational hypertension, gestational diabetes mellitus (GDM), miscarriage, congenital malformation, low birth weight, small-for-gestational-age (SGA) birth, preterm birth and caesarian delivery (15). Children born to mothers with asthma have a greater risk of developing this condition, yet evidence suggests that reducing maternal asthma exacerbations during pregnancy can mitigate this risk for children (16).

*Country Example:* In Australia, both midwives and doctors demonstrated under-confidence in management of asthma in pregnancy. Surveyed general practitioners expressed uncertainty in medical management of asthma during pregnancy, including reporting stopping or reducing doses of treatments even in well controlled cases (17). Midwives expressed in semi-structured interviews, that they were uncertain of their role in screening, counselling or managing patients with asthma during pregnancy (18). Despite the importance of optimal control of asthma in pregnancy on both the mother and fetus, there are few examples of specific screening or management programs for asthma in antenatal care provision. As is common in research, many studies on this topic exclude pregnant women (19).

Diabetes during pregnancy—both pregestational and gestational—increases the risk of pre-eclampsia, pre-term labor, and operative delivery, as well as increasing the risk of macrosomia, birth injury, and perinatal mortality (20, 21). Despite the significant impact of diabetes during pregnancy, screening, diagnosis, and the provision of care is often challenging in LMICs due to a lack of resources and shortage of trained personnel (20).

Perinatal mental health issues can persist through all stages of pregnancy, delivery, and the postnatal period and adversely affect maternal relationships with their infants and partners. In extreme cases, they may also lead to self-harm, a significant contributor to women’s deaths globally (22–24). In particular, maternal depression is associated with an increased risk of preterm labor and birth, intrauterine growth restriction, and pre-eclampsia (25).

*Country Example:* In Blantyre district, Malawi, it is estimated that 19% of women attending antenatal care services are suffering from antenatal depression (26). A further investigation demonstrated that introducing antenatal screening by midwives for maternal depression would be feasible to midwives working in this setting (27). The barrier to introducing screening was lack of a locally relevant screening tool, so the Screening Protocol for Prenatal Depression (SPADe) was developed, which was acceptable to surveyed midwives (27). However, mental health services in Malawi are underfunded and with limited or no access from antenatal clinics, so the team felt it was unethical to introduce the screening without effective treatment (27). This barrier needs to be addressed for effective scale up of this intervention (27).

Rarer NCDs diagnosed during pregnancy include cancers. These may hinder fetal growth, thereby increasing the risk of neonatal mortality, stillbirth, and infants being born small for gestational age (28).

#### Modifiable NCD risk factors and MNCH

Modifiable NCD risk factors refer to unhealthy behaviors that increase the risk of developing NCDs, such as tobacco use, harmful use of alcohol, physical inactivity, and an unhealthy diet. (29) These unhealthy habits also represent important risk factors for complications during pregnancy, fetal development and childbirth; which result in negative maternal, newborn, and child health outcomes (Box 1).

**BOX 1: Behavioural risk factors.**

**Smoking** during pregnancy has declined in recent years but remains surprisingly common although with wide variations across countries, from 5 to 40% (31). Smoking is a well-known risk factor for preterm birth and low birth weight. Other adverse pregnancy outcomes associated with maternal smoking include increased rates of miscarriage, ectopic pregnancies, prenatal hospitalization and congenital malformations (32, 33).

The global prevalence of **alcohol use** in pregnancy is estimated to be around 9.8%, with variations depending on the country. Alcohol exposure during pregnancy has been linked to stillbirth, impaired growth, preterm birth, hemorrhage in late pregnancy, premature rupture of membranes, postpartum hemorrhage, and fetal alcohol spectrum disorder in surviving infants with lifetime complications (34–36).

**Physical activity** during pregnancy is associated with decreased risks of adverse health outcomes, such as diabetes mellitus or gestational diabetes mellitus, pre-eclampsia/hypertensive disorders, and excessive weight gain. Physical activity during pregnancy is also a major contributing factor to improved psychological well-being and reduced risk of postpartum depression (29, 37). Nevertheless, studies show that a large proportion of pregnant women do not meet the WHO recommendations of moderate-intensity physical activity of at least 150 min per week for all adults between the ages of 18 and 64, and the time allocated to physical activity tends to decrease as pregnancies progress (38).

**Overconsumption and poor diet** are associated with excessive gestational weight gain, diabetes mellitus or gestational diabetes, hypertensive disorders, increased risk of large-for-gestational-age (LGA) births independent of maternal obesity, as well as negative impacts on long-term childhood growth (37, 39). Poor nutrition, particularly during pregnancy, is also linked to anemia. Women with anemia during the first or second trimesters are at heightened risk for having babies with low birth weight, preterm birth, and neonatal mortality (37, 40). Additionally, anemic women are more likely to die from postpartum hemorrhage, which is the leading cause of maternal death worldwide (41).

Household and ambient (i.e., outdoor) **air pollution** represent another important risk factor for NCDs, particularly ischemic heart disease, stroke, chronic obstructive pulmonary disease, and lung cancer. Air pollution is also a risk factor for complications during pregnancy and childbirth and is associated with negative maternal, newborn, and child health outcomes (40). Exposure to outdoor air pollution is increasing with global urbanization: currently, 55% of the world’s population lives in cities, and this percentage is expected to increase to 68% by 2050. Climate change is also affecting outdoor air pollutants (e.g., wildfire smoke). Additionally, the use of solid cooking fuels that produce household air pollution remains a common practice in many low- and middle-income countries (LMICs). Evidence indicates that outdoor air pollutants increase the risks of pregnancy hypertension, miscarriage, stillbirth, and preterm birth, and indoor air pollution has been associated with miscarriage, stillbirth, low birth weight and caesarean deliveries (42–46).

Pregnancy is a public health opportunity to address modifiable risk factors in a population that may not yet be experiencing their subsequent impacts on NCD development (30). The pregnant population also has a unique behavior change motivation that can be utilized by healthcare workers because of the impact of their behaviors on the development of their unborn child (30).

#### Metabolic NCD risk factors and MNCH

Metabolic NCD risk factors are commonly seen in MNCH. Almost 40% of adults are overweight or obese, which constitutes a tripling of the global obesity rate since 1975 (47). While the pathways leading to weight gain are complex, sedentary lifestyles and unhealthy diets largely consisting of (highly)processed foods high in energy, saturated fats, sugar, and sodium, are considered the major contributors. In 2016, more than 1.9 billion adults aged 18 years or older, or 39% of the world’s adult population, were considered overweight; of these individuals, more than 650 million (13%) were obese (47). As the global prevalence of overweight and obesity increases, the burden of associated NCDs continues to grow. The prevalence of obesity is two to three times higher in women and individuals of low socio-economic status across countries. Pregnant women are especially vulnerable (48).

Maternal overweight is now more common than underweight across all regions of the world. Being overweight and obese are important risk factors not only for diabetes but hypertension as well, and contribute to the onset of these conditions prior to and during pregnancy, with potential negative impacts on maternal, newborn, and child health (49, 50). Overweight and obese pregnant women face an increased risk of pre-eclampsia, gestational diabetes, higher rates of cesarean section deliveries, and longer hospital stays after giving birth. Infants born to overweight and obese mothers are also more likely to be born preterm, be born LGA, and require intensive hospital care at birth (51).

Hypertensive pregnancy disorders, namely pre-eclampsia, and eclampsia are major causes of maternal and perinatal deaths, preterm births, and low birth weight births. Hypertensive pregnancy disorders are also associated with higher risks of NCDs later in life (52). This link is currently underutilized and women could be put into NCD prevention and early management systems to screen for ongoing care. Similarly, high blood sugar levels cause complications in 17% of pregnancies, including for women with diagnosed and undiagnosed diabetes, and gestational diabetes mellitus (GDM) (53) and annual screening is recommended for type 2 diabetes following GDM due to future risk (14).

## Integrating care

While NCDs are directly influenced by a complex interplay of metabolic, behavioral (i.e., modifiable), and environmental risk factors, these diseases are deeply rooted in social, economic, and commercial determinants exacerbated by increased globalization and urbanization, resulting in unprecedented political and societal changes. To underscore the social rather than individual factors driving the NCD pandemic, some have even suggested reframing NCDs as “socially transmittable” conditions rather than “noncommunicable” (54).

The 2030 Agenda for Sustainable Development provides an opportunity for countries to re-emphasize not only improved survival, but also better health and well-being of mothers, newborns, and children, through increased investments and integrated and coordinated multisectoral and multistakeholder efforts. Studies suggest that a two-thirds reduction in MNCH deaths and a one-third reduction in premature NCD mortality will translate into 210,000 fewer maternal deaths and 690,000 fewer deaths among women of reproductive age, as well as reduce mortality by 2.4 million in women aged 50–69 years (55).

Pregnancy is an opportune time in the lives of women to diagnose and manage underlying conditions, including NCD risk factors and existing NCDs which can lead to adverse MNCH outcomes, thereby improving the health of women and their children across the life course (12, 56). This integrated approach is well articulated in the 2016–2030 Global Strategy for Women’s, Children’s, and Adolescents’ Health that provides the framework for continued investment in evidence-based actions, including addressing NCDs to end preventable maternal, newborn, and child mortality and morbidity (57). Box 2 defines integrated healthcare services and related concepts.

**Box 2: Health service integration (58, 59).**

- Integrated health services: Healthcare services that are managed and delivered so that people receive comprehensive health promotion, diseases prevention, diagnosis, treatment, management, rehabilitation, and palliative care services, coordinated across different levels and sites of care within and beyond the health sector, and according to people’s needs throughout the life-course. Integrated health services are integrated both clinically and functionally.
- Clinical integration: The coordination of patient care across a system’s different functions, activities, and operating units. It includes both horizontal and vertical integration:
- Horizontal integration: The coordination of patient care across the functions, activities, and operating units that are at the same stage/level of the service delivery process. Examples are consolidations, mergers, and shared services within a single level of care.
- Vertical integration: The coordination of patient care across the functions, activities, and operational units that are at different stages/levels of the service delivery process. Examples are the links between hospitals and medical groups, outpatient surgery centers, and home-based care agencies.
- Functional integration: The extent to which key support functions and activities such as financing, human resources, strategic planning, information management, marketing, and quality assurance/improvement are coordinated across all system units and levels.

We are concerned that the historic separation of NCH and MNCH care delivery is leading to missed opportunities to prevent and manage NCD risks factors, as well as worsening outcomes for existing NCDs that present in an MNCH setting. Priorising the integration of NCD and MNCH care, both in terms of functional delivery and clinical expertise, will mean that women and children receive a continuous, coordinated, and comprehensive service, that prevents and manages NCD risks as an integral part of their MNCH experience. This will help achieve the goal of early detection and management of NCDs across the continuum of care for mothers, neonates and children.

Developing an integrated approach to managing NCDs and MNCH aligns with WHO’s 13th General Programme of Work which calls for achieving UHC by improving access to integrated quality essential healthcare services and developing national packages that include NCDs along with MNCH interventions that go beyond the health system (6). However, guaranteeing the quality and consistency of health interventions when scaling up services to integrate NCD and MNCH care can be a challenge at many levels. Significant gaps in the provision of integrated delivery of health services can be identified, including at the levels of:

- Community: disease prevention, health promotion, health literacy, early identification, and seeking of care;
- Facility: multidisciplinary teams to ensure continuity of care, effective referral, disease management, and follow-up;
- Overall health system: supplies of medicines and technologies, diagnostics, logistics, programme management, and monitoring systems; and
- Policy and research.

As such, a wide range of strategies will be required for enabling health systems, especially at the community (primary care) level to deliver care encompassing interventions ranging from clinical to public health approaches to reduce exposure to risk factors through health promotion and disease prevention, to screen, diagnose and manage diseases, and to administer effective surveillance for monitoring risk factors and distribution of diseases.

## Contemporary challenges

Changing clinical practices in health service delivery is particularly complex. One of the major barriers to delivering integrated care is that health workers lack the training and resources to competently conduct the early detection of NCDs. Another important challenge is the shortage of specialized workforce (e.g., nurses, nutritionists, community workers) to form multidisciplinary teams needed to increase awareness and knowledge related to the prevention, self-management, and care of NCDs in communities, and to provide counselling on healthy lifestyles to women of reproductive age and their families. There is not currently a routine model for postnatal follow up for NCD related risk factors, modifiable behaviors or noted herald conditions that may be highlighted in pregnancy. Addressing health workforce-related barriers will require changes in the pre-service and in-service curriculum and training opportunities complemented by remote online education options (60).

Further challenges related to the delivery of integrated care for NCDs and MNCH include inconsistent global, regional and local guidelines; siloed protocols of care that do not adequately incorporate other services; outdated or unavailable national NCD guidelines for managing relevant chronic conditions before pregnancy and during antenatal and postpartum periods (e.g., screening and management of diabetes, hypertension, hyperlipidemia, cancer and mental health problems, as well as tobacco and alcohol screening and treatment). The lack of national capacity for evidence-based healthcare, including addressing NCDs to end preventable maternal, newborn, and child mortality and morbidity is a further critical issue in many contexts, as is limited access to essential medicines and basic equipment to diagnose NCDs (e.g., sphygmomanometers, glucometers and test strips, scales, measuring tape, cervical cancer screening tests). MNCH workers are increasingly expected to deliver not just NCD care, but HIV, TB, malaria, vaccinations, cervical cancer screening, pre-natal and post-natal services etc. that comes with a considerable training burden, need for easy access to multiple up-to-date guidelines. The requirement to take on additional work needs to be properly incentivized (61). Finally, many clinicians have limited experience in guideline implementation and clinical epidemiology. In practice, addressing these challenges will require the incorporation of quality improvement activities within healthcare teams, and the provision of ongoing education, training, and mentoring to healthcare workers.

## Framework for integration

The WHO Framework on integrated people-centered health services (WHA69.24) (58, 62) set out a vision of high quality health services that are coordinated across the continuum of care and are comprehensive, safe, effective, timely, efficient, and acceptable and all carers are motivated, skilled and operate in a supportive environment. The 69^th^ World Health Assembly endorsed five strategies to implement integrated care (63) that serve as a sensible framework for integrating NCDs and MNCH:

1. Engaging and empowering people and communities.
2. Strengthening governance and accountability.
3. Reorienting the model of care.
4. Coordinating services within and across sectors.
5. Creating an enabling environment.

De Jongh et al.’s (61) systematic review of studies that examined MNCH integration elicited several themes that serve for an integration framework; outlining the key components.

- Human resources.
  - Skills and training.
  - Guidelines.
  - Incentives.
  - Supervision, management and leadership.
- Medicines, supplies and technology.
- Financial resources.
- Information systems.

**Figure 1:**
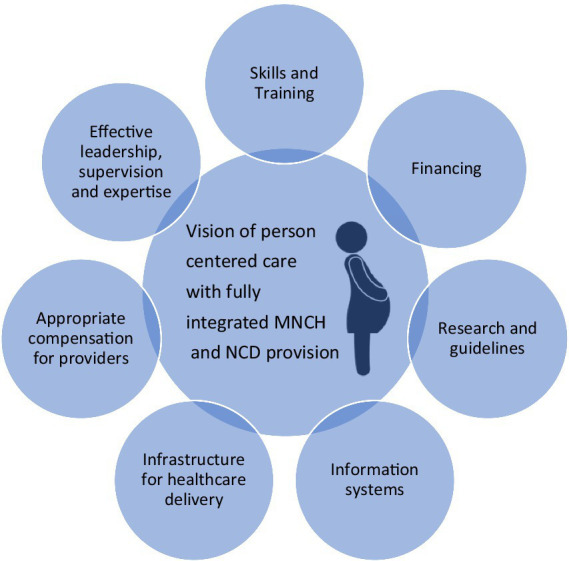
A framework for integration.

Integrated care is more sustainable, cost effective and satisfying for both patients and healthcare workers (64). However, delivering such changes to established services required significant political will as well as changes to guidelines and protocols, which take time and expertise which may not be available in all settings (64). Following childbirth, referral pathways into services for NCD prevention or management from MNCH care must be developed or strengthened. A preventative as well as curative approach should be adopted into care delivery pathways (64). Ideally, future care pathways will have NCD services fully integrated into MNCH care delivery across the continuum of care in a person centered approach. Examples of the essential components of a framework for an integrated, person centered approach, using the models set out by the World Health Assembly (58) and de Jongh et al. (61), is set out in Figure 1.

## Conclusion: converging agendas

Ultimately, jointly delivering NCD and MNCH services in a coordinated and integrated manner through UHC and primary health care (PHC) agendas is a political choice, which will be needed to undertake major reorientations of health systems to address the combined mortality and morbidity from noncommunicable, maternal, and neonatal diseases. The benefits of integrated care can only be realized if the historical, geopolitical, and socio-economic context is carefully considered, and the evolution of health systems, as well as demographic, epidemiological, and cultural factors, are taken on board through the involvement of all stakeholders.

The integrated approach to NCD and MNCH care will require scaling up not only priority interventions to address major gaps in the continuum of medical services at all levels, but intersectoral investments to improve geographic and financial access to care, including transport, roads, and emergency referral systems (65). There is a clear need to address the large evidence gap on the factors influencing integration. More rigorous studies involving comparisons between service delivery models in different socio-economic contexts will be important. Enhanced government commitment and resource allocation is needed to prioritize NCDs and improve the health of the current and future generations in all countries.

## Data availability statement

The original contributions presented in the study are included in the article/supplementary material, further inquiries can be directed to the corresponding author.

## Author contributions

SvA, AB, and TC conceptualized the manuscript. TC wrote the first draft of this manuscript. CLD and LNA developed the final draft of the manuscript. All authors revised the manuscript for important intellectual content and approved the final version.

## Conflict of interest

The authors declare that the research was conducted in the absence of any commercial or financial relationships that could be construed as a potential conflict of interest.

## Publisher’s note

All claims expressed in this article are solely those of the authors and do not necessarily represent those of their affiliated organizations, or those of the publisher, the editors and the reviewers. Any product that may be evaluated in this article, or claim that may be made by its manufacturer, is not guaranteed or endorsed by the publisher.

## Author disclaimer

The authors alone are responsible for the views expressed in this article and they do not necessarily represent the decisions, policies or views of the World Health Organization.
